# Method for the differentiation of radiation-induced photocurrent from total measured current in P3HT/PCBM BHJ photodiodes

**DOI:** 10.1016/j.mex.2020.101125

**Published:** 2020-11-04

**Authors:** Michael A Hupman, Irina Valitova, Ian G Hill, Alasdair Syme

**Affiliations:** a Department of Physics and Atmospheric Science, Dalhousie University, Halifax, Nova Scotia B3H 4R2, Canada; b Department of Radiation Oncology, Dalhousie University, Halifax, Nova Scotia B3H 4R2, Canada; c Department of Medical Physics, Nova Scotia Health Authority, QEII Health Science Centre, Halifax, Nova Scotia B3H 1V7, Canada

**Keywords:** Electrode irradiation, Compton current, Photocurrent contamination

## Abstract

Thin film radiation-detecting diodes fabricated in the laboratory, such as an organic bulk heterojunction, often contain conductive leads, indium tin oxide traces and metallic interconnects which are exposed to the high-energy photon beam during operation. These components generate extraneous radiation-induced currents, that, if not accounted for, will erroneously be attributed to the detector. In commercial devices, these contributions are mitigated by minimizing the size of these components, an approach that is often not feasible in a research lab. Here we demonstrate a method to measure these extraneous signals, and by subtraction, correct the gross signal to accurately reflect the signal generated in the active volume of the diode itself.

The method can effectively correct the extraneous signal.

The method showed promise over a range of photon beam energies, dose rates, and field sizes.

## Graphical abstract

**Figure:**
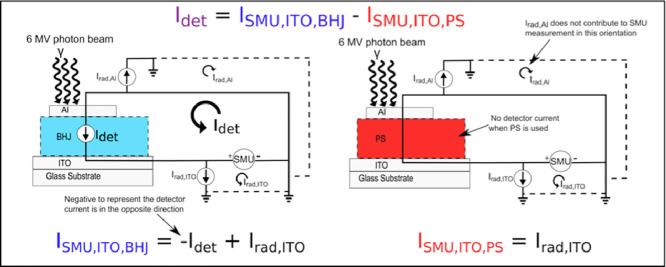

**Specifications Table**

**Table:** 

Subject Area:	Physics and Astronomy
More specific subject area:	Organic electronic devices, radiation detection
Method name:	Differentiation of Radiation-Induced Photocurrent from Total Measured Current in BHJ Photodiodes
Name and reference of original method:	P3HT/PCBM fabrication Process [1], [2], [3]
Resource availability:	Website of Keithley source measurement units (SMU): https://www.tek.com/keithley-source-measure-units Website of Varian's Truebeam: https://www.varian.com/products/radiotherapy/treatment-delivery/truebeam Website for Sun Nuclear Corporation solid water: https://www.sunnuclear.com/products/solid-water-he

## Methods details

*Method details

## Introduction

The ability to accurately measure absorbed dose (energy deposited per unit mass) is a critical prerequisite for the safe and effective use of ionizing radiation to treat cancer. Numerous types of devices can be calibrated for this purpose (e.g. ionization chamber, thermoluminescent dosimeter, silicon diode, film, etc.); in this work, we describe measurements in radiation fields using a P3HT/PCBM bulk heterojunction (BHJ) photodiode. Organic semiconductor-based dosimeters have gained interest because of their low atomic number, which is comparable to that of human tissue, which suggests that these devices could require fewer or smaller correction factors to translate a measurement into absorbed dose in comparison to their silicon counterparts.

In an ideal radiation detector, the entirety of the measured signal is generated in the “active volume” of the detector. In the case of thin film diodes, such as organic BHJ photodiodes, this would be in the BHJ itself (i.e. the semiconductors), between the two electrodes. In the case of electrical devices, however, an additional current can be generated due to interactions in the electrodes, connectors, and wires. This “Compton current” constitutes an extraneous signal that contaminates the true signal of interest and can detract from the accuracy of a dose measurement.

## Device fabrication

Fabrication of the organic P3HT (Molecular weight 50–80 kg/mol, Brilliant Matters, Quebec City, Canada)/PCBM (Solenne BV, Groningen, Netherlands) BHJ diodes followed established methods from the photovoltaic literature [1], [2], [3], [4]. ITO-coated glass substrates were patterned by etching with hydrochloric acid for approximately 5 min. The etched slides were cleaned by sonicating for 15 min in successive solutions of deionized water with Sparkleen (Thermo Fisher Scientific, Waltham, USA), deionized water, acetone, and ethanol followed by UV-ozone for 20 min. PEDOT:PSS (Clevios P VP Al 4083, Heraeus, Hanau, Germany) was spin coated for 60 s at 5000 rpm and placed on a hot plate at 150 °C for 20 min in air. Equal parts P3HT and PCBM in chlorobenzene (Sigma-Aldrich, St. Louis, USA) were spin coated in inert atmosphere (N_2_) inside a glove box with different recipes to create devices of three different thicknesses. A 2% by weight solution was spin coated at 2000 rpm for 60 s resulting in a film ≈100 nm. A 2% by weight solution was spin coated for 60 s at 100 rpm, 60 s 500 rpm, and then 60 s at 1000 rpm resulting in a film ≈200 nm. A 4% by weight solution was spin coated with the same speeds as the ≈200 nm film resulting in *a* ≈ 420 nm film. Film thickness was measured with a Dektak 8 stylus profilometer (Bruker Corporation, Billerica, USA). The films were annealed at 110 °C for 1 hour in inert atmosphere (N_2_). Aluminum was deposited with vacuum thermal evaporation at a rate of ≈1 Å /s to a thickness of ≈80 nm defining an active area of 3.25 mm^2^. The diodes were encapsulated with an epoxy (Ossila, Sheffield, UK), which was cured under UV-light for 20 min.

As a control, devices were made with a polystyrene (PS) (Molecular weight ≈280 kg/mol, Sigma-Aldrich, St. Louis, USA) layer in place of the P3HT/PCBM semiconductors with the rest of the device following the fabrication detailed above. Solutions of 3, 6, and 10% by weight solution of PS in toluene were spin coated at 2000 rpm resulting in films ≈110 nm, ≈220 nm, and ≈390 nm, respectively.

## Extraneous signal

**Fig. 1:**
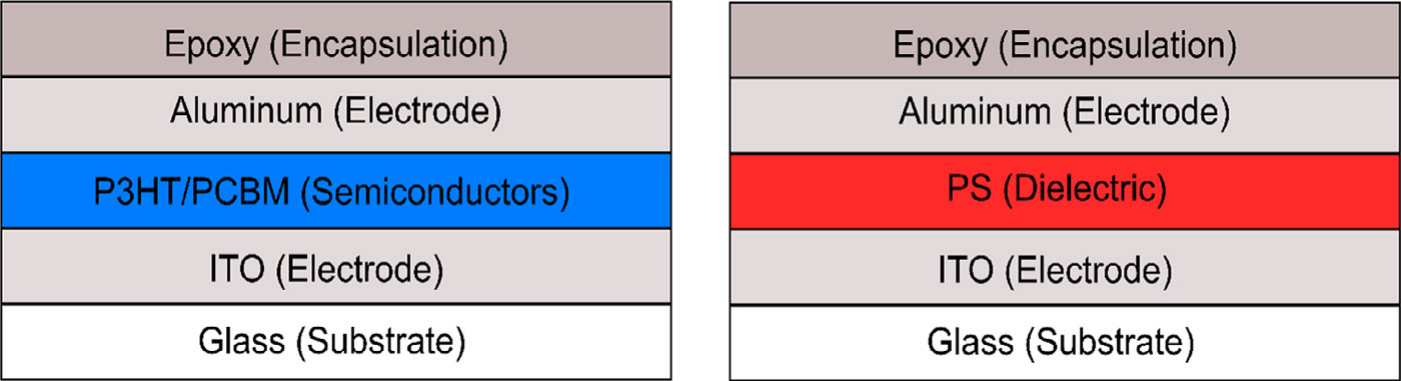
Schematic diagrams of the BHJ photodiodes (left) and PS control devices (right). The devices are identical except the organic semiconductors are replaced by PS in the control devices. Care was taken to create recipes that produced PS layer thicknesses that matched BHJ semiconductor thicknesses as much as possible.

The BHJ photodiode was exposed to high energy (megavoltage) photons from a Varian TrueBeam medical linear acceleartor (Varian Medical Systems, Palo Alto, USA). A schematic of the BHJ photodiode is shown in Fig. 1a. Initial measurements used one SMU with the positive output connected to the ITO electrode, and negative connected to the aluminum electrode. Our hypothesis was that the high energy irradiation would create excitons in the BHJ layer similarly to the way in which excitons are produced when the BHJ is exposed to visible light. If that were true, holes would migrate to the ITO electrode, resulting in a measurement of a negative current. However, what we measured was a positive current. To investigate the problem further we used a two-SMU setup which measured the current coming from each electrode independently, and simultaneously. If our initial hypothesis was correct, electrons would migrate to the aluminum electrode and a positive current would be measured of equal magnitude to the negative current on the ITO electrode. However, the currents measured on the two electrodes were both positive, and of different magnitudes. We hypothesized that an undesirable current was induced in the electrodes, wires, etc. due to the shower of electrons induced by the radiation beam. To correct for this Compton current we needed a device which would measure the Compton current without any radiation-induced photocurrent in the BHJ. For this purpose we fabricated a device with polystyrene (PS) in place of the BHJ as shown in Fig. 1b. A PS device should have negligible photocurrent because without a donor/acceptor interface there should be no dissociation and, even if free charges were generated, the mobility of these charges would be very low in PS, resulting in a very low extraction efficiency. An important detail of the measurement setup for this correction technique is to ensure an identical geometry of the electrodes, wires, etc. with respect to the radiation field for both device types to ensure the Compton current will be the same in both measurements.

## Correction method

**Fig. 2:**
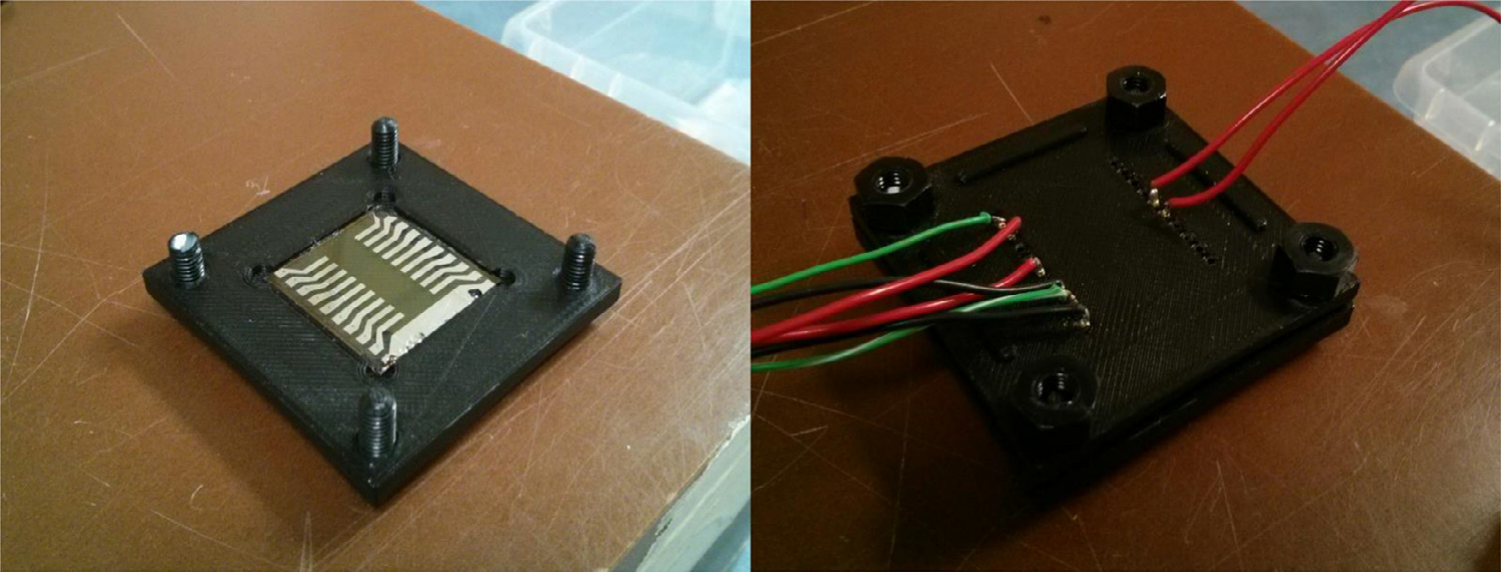
Picture of the device holder with and without the top to allow easy electrical contact. The holder shields light from reaching the photodiode and allows easy and consistent contact on the devices.

Initially, to measure the current coming from the device, triaxial cables were used which had alligator clips at the end to connect to contact pads on the edge of the substrate. To decrease the Compton current, the bulky alligator clips needed to be placed outside of the irradiation field. To accomplish this a 3-D printed holder was used with pogo pins to contact the photodiode (Fig. 2). Next, wires were soldered to the ends of the pogo pins and brought outside of the beam. This reduced the size of the contacts in the beam. Furthermore, the 3-D printed holder allowed for easy device alignment within the beam and the path of the wires could be more easily reproduced when changing from a BHJ diode to a PS device. An added benefit of the 3-D printed holder is that it makes it easy to shield the BHJ photodiode from light.

**Fig. 3:**
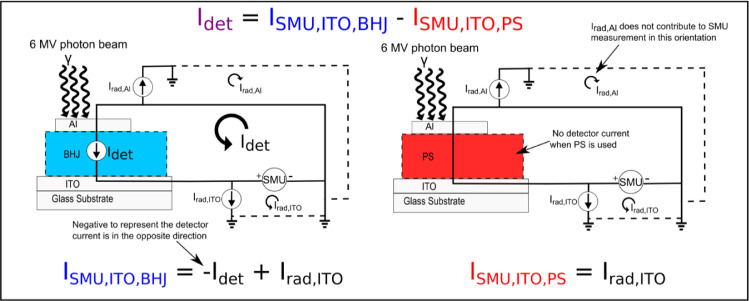
Schematic showing the two circuits used for the correction method. On the left the bulk heterojunction (BHJ) diode is placed in the radiation beam and the current is measured with an SMU connected to the ITO electrode. This measurement has current induced in the detector (I_det_) and Compton current induced in the electrodes and wires (I_rad,ITO_). On the right a polystyrene device has been placed in the irradiation beam in place of the BHJ diode and the current measured coming from the ITO electrode. The PS device will have no detector current (I_det_), but will measure a Compton current (I_rad,ITO_). To calculate the detector current (I_det_) the current measured with the PS setup is subtracted from the BHJ diode setup.

A BHJ photodiode was irradaited and the current measured on the ITO electrode using an SMU (Keithley 2614B, Tektronix Inc., Beaverton, USA) as depicted in Fig. 3. Two distinct currents are labelled that contribute to the measured current. First, there is the current induced inside the BHJ semiconductors I_det_ which is analogous to photocurrent generated when a light is incident on a photodiode. Second, there is a Compton current I_rad,ITO_ which is generated in the electrodes, contacts, and wires associated with the ITO electrode due to the incident radiation beam and the associated secondary electrons impinging upon them. A similar current, I_rad,Al_, is generated in the top-contact electrode and associated conductors. When a megavoltage photon beam (of the type encountered in a radiation therapy setting) interacts in a water-like material it creates electrons primarily via Compton scatter and some photoelectric effect. These electrons have enough energy to produce additional excitations and ionizations. The net result is a shower of electrons spanning a large range of energies. Electrons interacting in the electric contacts and wires can produce a current that contributes to the measured current represented by I_rad,[ITO/Al]_ in the figure. The Compton current produced in the aluminum contact and wires I_rad,Al_ does not contribute to the current measured on the ITO electrode. However, if the SMU were connected to measure the current coming from the aluminum electrode I_rad,Al_ will contribute to the current and I_rad,ITO_ will not. Furthermore, the magnitude of I_det_ will be the same, but it will be the opposite direction. In equation form these measurements can be represented by:

I_SMU,ITO,BHJ_ = -I_det_ + I_rad,ITO_

I_SMU,Al,BHJ_ = I_det_ + I_rad,Al_

Where I_SMU,[ITO/A_*_l_*_],BHJ_ is the current measured by the SMU when connected to the BHJ photodiode to the ITO/Al electrode. Since I_det_ is negative when measuring the ITO current, the Compton current direction will oppose it (given the current directions defined in Fig. 3). The magnitude of I_rad,[ITO/Al]_ will depend on the area of the electrodes and the amount of connector and wires in the irradiation beam (as well as beam parameters such as energy, dose rate, etc.).

To correct for the I_rad,[ITO/Al]_ current and obtain the detector current I_det_ we fabricated devices with polystrene (PS) instead of the BHJ. We expect that excitons will be generated in the PS layer just like the BHJ. However, given the insulating nature of PS, and the absence of a donor/acceptor interface, we do not expect any significant detector current. The measured current will be dominated by the extraneous contributions. Furthermore, if the irradiation setup is the same as when irradiating the BHJ (same beam energy, dose rate, etc.) then the I_rad,[ITO/Al]_ current should be the same as measured for the BHJ. By measuring this current and subtracting it from the current measured with the BHJ measured current we can calculate the I_det_ current with the ITO electrode using:

I_SMU,ITO,BHJ_ - I_SMU,ITO,PS_ = (-I_det_ + I_rad,ITO_) - (I_rad,ITO_)

I_SMU,ITO,BHJ_ - I_SMU,ITO,PS_ = -I_det_

Or on the aluminum electrode:

I_SMU,Al,BHJ_ - I_SMU,Al,PS_ = (I_det_ + I_rad,Al_) - (I_rad,Al_)

I_SMU,Al,BHJ_ - I_SMU,Al,PS_ = I_det_

To do this correction only one SMU is needed. The current can be measured from the same electrode on the BHJ device and the PS device and the current subtracted. An additional check to increase confidence in the correction method can be performed by simultaneously measuring the current with both electrodes and comparing the calculated detector current for both direction and magnitude.

### Validation of method and quantification of Compton current

**Fig. 4:**
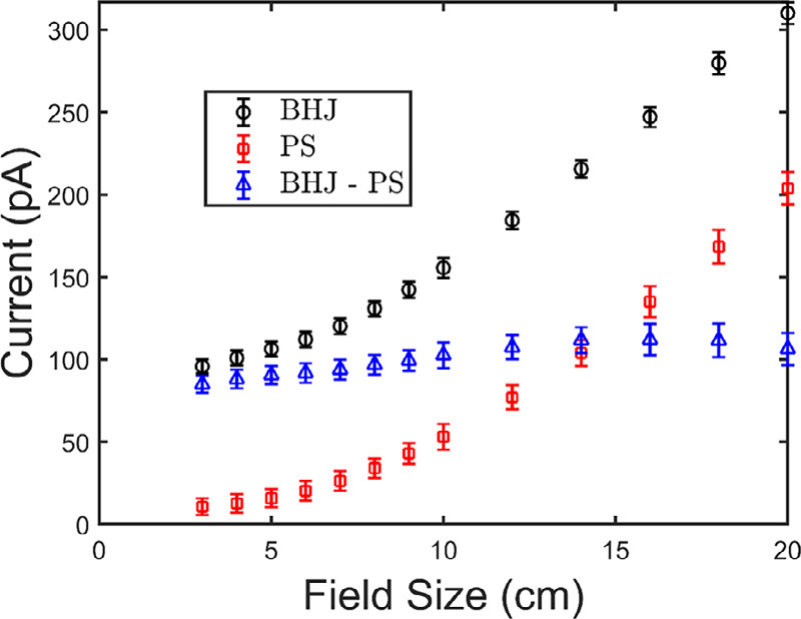
The current is plotted as a function of the field size for the bulk heterojunction (BHJ) photodiode, polystyrene (PS) device, and the corrected current (PS current subtracted from BHJ). For both the BHJ and PS the current increases with field size due to increasing the amount of wires irradiated. Data in this figure were used to produce figure 9 in Hupman et al. (2020) [4].

**Fig. 5:**
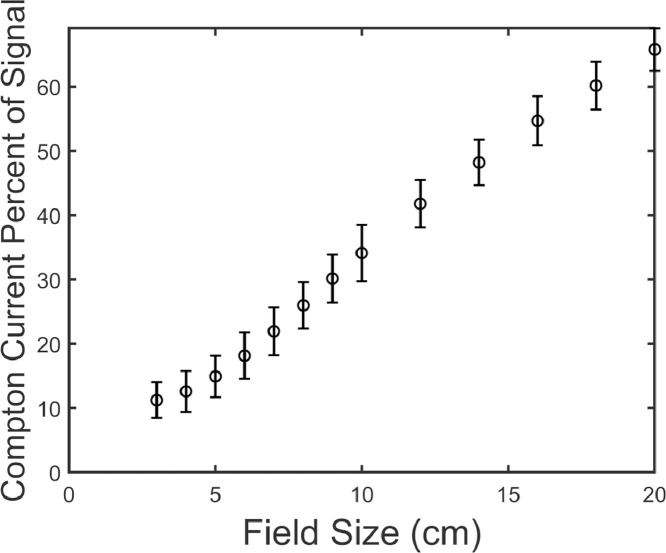
The percent of the total current comprised of Compton current (PS current divided by BHJ current from Fig. 4) is plotted as a function of field size. As the field size increases the percentage of the signal comprised of the Compton current increases.

**Fig. 6:**
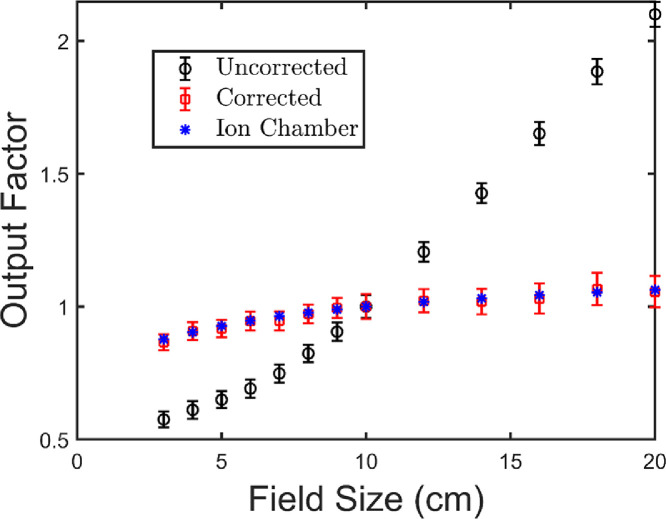
The output factor (detector response normalized to a 10 × 10 cm^2^ field size) was measured for a 6 MV photon beam and compared to ion chamber measurements. The current is normalized to the signal measured with a 10 × 10 cm^2^ field size. After the correction there is good agreement with the ion chamber. Figure taken from Hupman et al. (2020) [4].

Fig. 4 shows the current measured as a function of field size for the BHJ photodiode, PS device, and the corrected current (subtract the PS measured current from the BHJ measured current). For comparison measurements were done with no device attached to the leads and the alligator clips placed in the beam. Under these conditions 200 ± 50 pA was measured for a 10 × 10 cm^2^ field which is more than the induced current in a BHJ diode. The figure shows that the Compton current (PS device) contributes a significant portion of the total signal (BHJ diode). Furthermore, the proportion of the total signal contributed due to Compton current increases with field size, as the leads receive a larger dose. This is shown in Fig. 5 more explicitly, where the percent of the total signal due to Compton current is given as a function of field size. Compton current makes up 11 ± 3% of the total signal for a field size of 3 × 3 cm^2^, but contributes over 60% of the signal for a 20 × 20 cm^2^ field. Compton current increases with field size because more length of wire is exposed to the beam. The corrected current increases with field size as well because more scatter is present which increases the dose at the middle of the field. This effect is well-known and has been verified using a commercial ionization chamber (Fig. 6). Fig. 6 shows output factors which are the response of the detector normalized to the response for a 10 × 10 cm^2^ radiation field. Output factors are shown for the BHJ before and after correction compared to a commercial ion chamber. After correction, the data match the ion chamber well, demonstrating the validity of our correction method. Furthermore, this example emphasizes the importance of the device and substrate contacts, the sample holder (and pogo pins), the size and length of connecting wires, and geometry of the irradiation field on the magnitude of the Compton current contribution.

### Signal change with device thickness

**Fig. 7:**
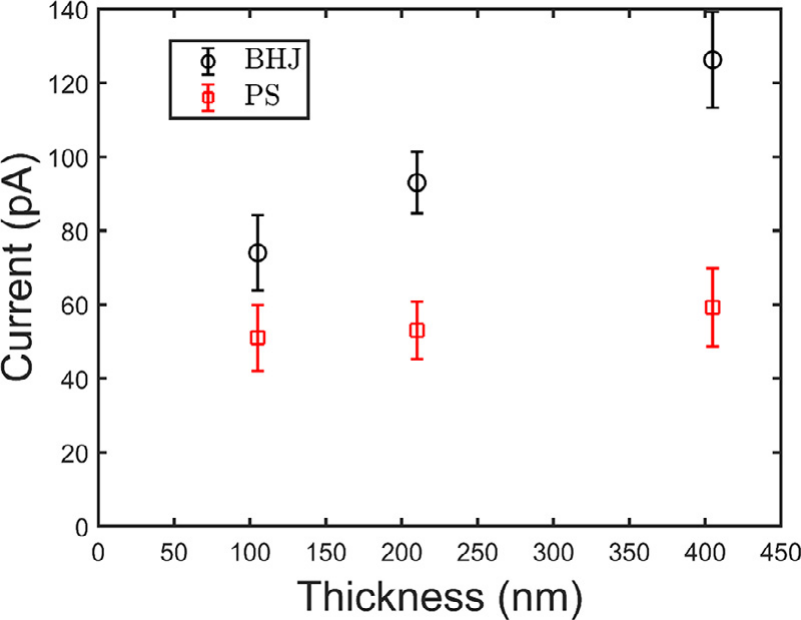
Current measured for three different thicknesses for the BHJ diode and the PS device. The BHJ diode shows an increase of current with thickness. The PS device shows no significant change in current with thickness. Data from Fig. 7 of Hupman et al. (2020) were included in this figure for context [4].

Fig. 7 shows the current of BHJ diodes and the PS devices as a function of thickness. For the PS device there is no significant change with thickness because the signal is dominated by Compton current from the contacts, wires, etc. which does not change with PS thickness. The BHJ diode shows a significant increase in signal with thickness of device.

### Suggestions to reduce Compton current

As demonstated this correction technique has shown promising results. However, it is best to reduce the Compton current contribution as much as possible before implementing the technique. It is important to minimze the footprint of the electrodes, contacts, and wires where possible (this could include methods such as wire bonding for connections, and using the smallest gage wires possible). Replacing ITO with organic conductors may reduce the contribution of the Compton current. The orientation of the detector should be chosen to reduce the footprint of the wires in the beam.

## Conclusion

In conclusion, we have presented a technique to correct the extraneous signal contributed due to interactions in the electrodes and wires when a thin-film photodiode is exposed to irradiation. The field size dependence shows that the magnitude of the problem depends on the length of wires placed within the irradiation beam. After using the correction technique the signal due to photocurrent originating within the BHJ agreed well with ion chamber measurements.

## Declaration of Competing Interests

The authors declare that they have no known competing financial interests or personal relationships that could have appeared to influence the work reported in this paper.
